# Multiannual effects of induced plant defenses: Are defended plants good or bad neighbors?

**DOI:** 10.1002/ece3.4365

**Published:** 2018-08-14

**Authors:** Rafael Fonseca Benevenuto, Stein Joar Hegland, Joachim Paul Töpper, Knut Rydgren, Stein R. Moe, Cesar Rodriguez‐Saona, Tarald Seldal

**Affiliations:** ^1^ Faculty of Engineering and Science Western Norway University of Applied Sciences Sogndal Norway; ^2^ Faculty of Environmental Sciences and Natural Resource Management Norwegian University of Life Sciences Ås Norway; ^3^ Norwegian Institute for Nature Research Bergen Norway; ^4^ Department of Entomology Rutgers University New Brunswick New Jersy

**Keywords:** bilberry, boreal forest, delayed response, methyl jasmonate, multiannual effects, plant–plant interactions, *Vaccinium myrtillus*

## Abstract

Defenses induced by herbivore feeding or phytohormones such as methyl jasmonate (MeJA) can affect growth, reproduction, and herbivory, not only on the affected individual but also in its neighboring plants. Here, we report multiannual defense, growth, and reproductive responses of MeJA‐treated bilberry (*Vaccinium myrtillus*) and neighboring ramets. In a boreal forest in western Norway, we treated bilberry ramets with MeJA and water (control) and measured responses over three consecutive years. We observed the treatment effects on variables associated with herbivory, growth, and reproduction in the MeJA‐treated and untreated ramet and neighboring ramets distanced from 10 to 500 cm. MeJA‐treated ramets had fewer grazed leaves and browsed shoots compared to control, with higher effects in 2014 and 2015, respectively. In 2013, growth of control ramets was greater than MeJA‐treated ramets. However, MeJA‐treated ramets had more flowers and berries than control ramets 2 years after the treatment. The level of insect and mammalian herbivory was also lower in untreated neighboring ramets distanced 10–150 cm and, consistent with responses of MeJA‐treated ramets, the stronger effect was also one and 2 years delayed, respectively. The same neighboring ramets had fewer flowers and berries than untreated ramets, indicating a trade‐off between defense and reproduction. Although plant–plant effects were observed across all years, the strength varied by the distance between the MeJA‐treated ramets and its untreated neighbors. We document that induced defense in bilberry reduces both insect and mammalian herbivory, as well as growth, over multiple seasons. The defense responses occurred in a delayed manner with strongest effects one and 2 years after the induction. Additionally, our results indicate defense signaling between MeJA‐treated ramets and untreated neighbors. In summary, this study shows that induced defenses are important ecological strategies not only for the induced individual plant but also for neighboring plants across multiple years in boreal forests.

## 1 INTRODUCTION

Plants have evolved a diversity of structural, constitutive, and inducible defenses to protect tissues, seeds, and fruits from attacking herbivores, fungi, and pathogens (Agrawal, 1999). Green and Ryan (1972) were the first to demonstrate that plants under attack from herbivores produce chemical defense compounds that help to protect them from further damage. For example, the emission of volatile organic compounds (VOCs) functions as warning signals to deter herbivores and attract beneficial predatory insects (Dicke, 1999; Macel & Vrieling, 2003; Melis et al., 2006; Nieminen, Suomi, Van Nouhuys, Sauri, & Riekkola, 2003; Paré & Tumlinson, 1999). Moreover, plant VOCs are key signals in plant–plant interactions, and work as external signals in the activation of plant defense systems (Arimura et al., 2002; Dolch & Tscharntke, 2000; Heil & Karban, 2010; Karban, 2001; Ruther & Kleier, 2005). Plant–plant interactions can occur both above and belowground and are mediated through internal signals driven by specific compounds moving within interconnected ramets in clonal species (Gómez, Van Dijk, & Stuefer, 2010), or by external VOCs emitted by neighboring plants (Rodriguez‐Saona, Rodriguez‐Saona, & Frost, 2009). The role of these VOCs is mainly regulated by the hormone jasmonic acid (JA) and related compounds, which perform a key role in the activation of plant defense responses (Staswick & Lehman, 1999; Wasternack et al., 1998). Laboratory and field studies have shown that plant chemical defense systems can be elicited experimentally by exogenous application of methyl jasmonate (MeJA), a VOC derivate of jasmonic acid, known as an omnipresent defense signal in plants (Koo & Howe, 2009; Pieterse, Van der Does, Zamioudis, Leon‐Reyes, & Van Wees, 2012).

In the boreal forest, bilberry (*Vaccinium myrtillus* L.) is a key food plant for many insects, birds, and mammals (Hjältén, Danell, & Ericson, 2004; Jacquemart, 1993; Selås, 2001; Welch, Keay, Kendall, & Robbins, 1997). As a result of its ecological importance, bilberry is an ideal organism for studies on inducible plant defense responses, as well as plant–plant and plant–animal interactions under natural field conditions. In previous studies, defenses induced by herbivore feeding or MeJA treatment were shown to reduce herbivory and increase reproduction of the damaged or treated bilberry plants (Hegland, Seldal, Lilleeng, & Rydgren, 2016; Seldal, Hegland, Rydgren, Rodriguez‐Saona, & Töpper, 2017). However, little is known from natural systems about the multiannual effects of induced plant defenses. Similarly, the extent to which such effects are transferred to neighboring plants is unknown (Karban, Ishizaki, & Shiojiri, 2012; Karban & Maron, 2002). Such studies may improve our understanding of the ecological consequences of induced defenses and plant–plant interactions on herbivore population dynamics.

Over three consecutive years, we explored plant defense activation in response to exogenous MeJA application in bilberry and its effects on untreated neighboring bilberry ramets under natural field conditions. Inducible plant defense responses are assumed to be energetically costly due to the allocation of resources from growth and reproduction to defense (Karban, Yang, & Edwards, 2014; Rodriguez‐Saona, Polashock, & Malo, 2013; Sampedro, Moreira, & Zas, 2011; Seldal et al., 2017). Thus, in the first year after treatment, we predicted decreased insect and mammalian herbivory and reduced plant size (growth) and reproduction in MeJA‐treated bilberry ramets compared to untreated controls (prediction I). Based on the role of VOCs in the detection of induced defenses in neighboring plants (Arimura et al., 2002; Dolch & Tscharntke, 2000; Farag & Pare, 2002; Hare, 2011; Heil & Karban, 2010; Karban, 2001; Karban, Baldwin, Baxter, Laue, & Felton, 2000; Ruther & Kleier, 2005), we also predicted less herbivory and reduced growth and reproduction of untreated neighboring ramets at short distances from the induced plant (i.e. 10–500 cm; prediction II). Finally, because bilberry is a relatively slow‐growing perennial and deciduous shrub (Flower‐Ellis, 1971; Jacquemart & Thompson, 1996; Ritchie, 1956), we predicted a 1‐year delay of the largest resource allocation effects, and possible long‐term (multiannual) reduction in growth, reproduction, and insect and mammalian herbivory of MeJA‐treated and untreated neighboring ramets (prediction III) (Haukioja, Suomela, & Neuvonen, 1985; Zvereva, Kozlov, Niemelä, & Haukioja, 1997).

## 2 MATERIAL AND METHODS

### 2.1 Study system

We conducted a study of induced plant defense from 2013 to 2015 in a ca. 20‐year old 50 × 200 m clear‐cut in a pine forest at 350 m above sea level. The study area, Kaupanger in western Norway (61.2°N, 007.2°E), has annual precipitation of 700–900 mm and a mean summer temperature range of 12–16° C (Moen, 1999). Pine (*Pinus sylvestris* L.), bilberry (*V. myrtillus*), lingonberry (*Vaccinium vitis‐idaea* L.), and crowberry (*Empetrum nigrum* L.) are the most abundant plant species in the field layer. The area has a dense winter population of red deer (*Cervus elaphus* L.), which is the most abundant wild ungulate in Norway (pers. obs. S.J. Hegland). Bilberry, our study species, is a long‐lived deciduous clonal dwarf shrub, with evergreen stems usually 10–60 cm high (Flower‐Ellis, 1971; Ritchie, 1956). Although we do not have specific information regarding clone size and distribution for the study area, we have based our work on the assumption that rhizomes can reach around 200 cm in length, depending on age, and the proportion of genetic variation within population is high (Albert, Raspé, & Jacquemart, 2003, 2004; Flower‐Ellis, 1971). Bilberry is also a key species in boreal and alpine ecosystems because of its ecological role as a food source for many invertebrate and vertebrate species (Dahlgren, Oksanen, Sjödin, & Olofsson, 2007; Hegland, Jongejans, & Rydgren, 2010). The main mammalian herbivores feeding on bilberry in the study area are red deer and various rodent species, whereas the most common insect herbivores are Geometridae larveae (pers. obs. S.J. Hegland). Bumblebees, honeybees, and syrphid flies are the main pollinators for this species (Jacquemart, 1993; Jacquemart & Thompson, 1996).

### 2.2 Experimental design and treatments

**Figure 1:**
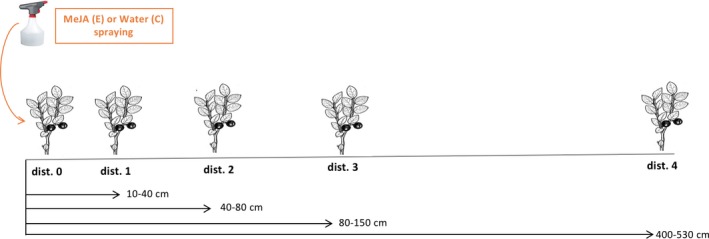
Transect design with the distances between the treated bilberry ramet and its untreated neighbors. C: control transect; E: experimental transect; MeJA: methyl jasmonate [Colour figure can be viewed at http://wileyonlinelibrary.com]

In June 2013, we established ten blocks of 350 m^2^ (10 m × 35 m), leaving a minimum of ten meters between each block to avoid interaction. To reduce variation in light conditions, humidity, and snow cover, we established the ten blocks oriented in the same direction in a uniform clear‐cut facing southwest. Within each block, four transects were established at least ten meters apart with five individually marked bilberry ramets in each, ranging in height from 10 to 25 cm. The five ramets were located 10–40 cm (dist. 1), 40–80 cm (dist. 2), 80–150 cm (dist. 3), and 400–530 cm (dist. 4) from ramet one (dist. 0) in each of the transects (Figure 1). Transects were subsequently randomly assigned and exposed to two treatments with two replicates in each block. In 2013, we treated the first ramet (dist. 0) in each transect with either 10 mM MeJA (experimental transects) or water (control transects). Spraying was repeated three times within 2 weeks in the first year (2013). Prior to treatment application, MeJA was diluted with 95% (v/v) ethanol and then with water to provide 10 mM MeJA (Seldal et al., 2017). To avoid rapid evaporation of MeJA, a wad of cotton wool was attached to the stem of the ramet at ground level and saturated with the assigned treatment until the point of runoff (Seldal et al., 2017). The ramets were not exposed to further treatments in 2014 and 2015 to evaluate possible multiannual effects on growth, reproduction, and herbivory. We treated only the first ramet in each transect to evaluate possible effects of plant–plant interaction between MeJA‐treated and its untreated neighboring ramets.

### 2.3 Sampling procedure

**Figure 2:**
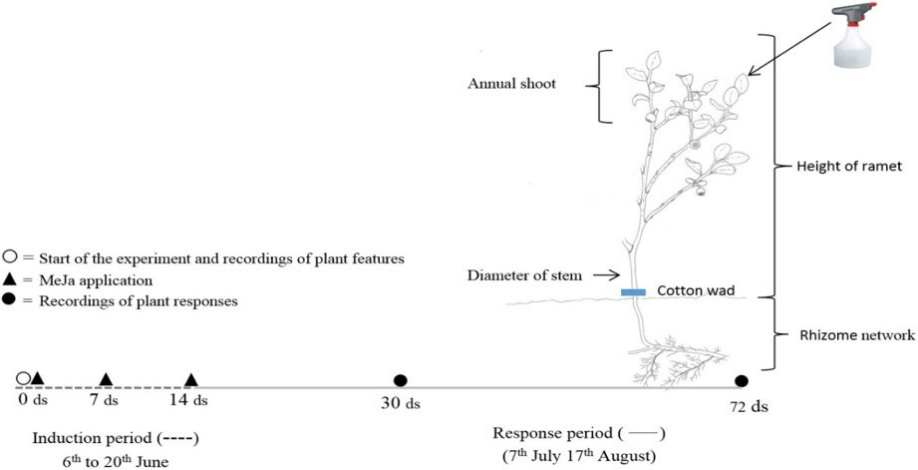
A bilberry ramet including size recordings, and timeline for the induction and response period when the measurements were recorded. ds: days MeJA: Methyl jasmonate [Colour figure can be viewed at http://wileyonlinelibrary.com]

Before the start of the MeJA treatments (6 June, 2013), we measured ramet height from the ground to crown with a ruler and stem diameter at ground with digital calipers. We also counted the number of annual shoots, flowers, leaves, browsed shoots, and leaves grazed by chewing insects (Figure 2). We repeated recordings of these variables 30 and 72 days after the initial treatment in 2013. In these subsequent recordings, we also counted berries. We repeated the measurements in 2014 and 2015. Plant height (*H*), stem diameter (DS), and number of shoots (AS) were used to calculate dry mass (DM) of each ramet as a nondestructive estimation of plant size using the formula of Hegland et al. (2010): log_2_(DM) = 1.41700 × log_2_(DS) + 0.97104 × log_2_(*H*) + 0.44153 × log_2_(AS + 1) − 7.52070.

### 2.4 Data analysis

We analyzed how exogenous MeJA application of bilberry ramets affected growth (dry mass), reproduction (number of flowers and berries), and herbivory (ratio of grazed leaves by insect herbivores; number of browsed shoots by large herbivores) by comparing untreated control ramets at dist. 0 with corresponding ramets in the experimental transects (MeJA dist. 0 = MeJA‐treated ramets; MeJA dist. 1–4 = untreated ramets with increasing distances from the treated ramet). For each response variable, we parameterized a generalized linear mixed‐effects model under Bayesian inference with intercepts and seasonal time slopes (three seasonal censuses) for each treatment (control, MeJA dist. 0–4) in every year (2013–2015), that is, we adopt a “means parameterization” approach (see also Section 2.4.2 for interpretation) (Kéry, 2010). In these models, differences in intercepts between control and MeJA treatment ramets represent differences in the respective responses at the first census in each year (intercept effect). Differences in slopes reflect differences in the temporal development during the season (slope effect). Effects on reproduction (flowers and berries) were analyzed yearly, based on records from the last census in each year. All models included random intercepts for each individual to account for the repeated measurements through time. For models of dry mass, we used a Gaussian error distribution with an identity link; for models of “ratio of insect grazed leaves,” we used a binomial error distribution with a logit‐link; and for models of “number of browsed shoots,” “number of flowers,” and “number of berries,” we used a Poisson error distribution with a log‐link. The binomial and Poisson models were specified to account for overdispersion by extending the error structure with an observation‐level random intercept effect (modeling of errors drawn from a normal distribution extra to the implicit deviation in the Poisson family). Furthermore, the Poisson models were specified to account for zero‐inflation (did not apply to flower/berry models) by modeling an observation‐level Bernoulli process (Kéry, 2010). Upon inclusion of “block” as a random effect, the models failed to converge, which likely was due to increasingly uneven sampling size in 2014 & 2015 following the death of some plants. Therefore, we decided to focus on modeling individual random effects, overdispersion, and zero‐inflation. All models were run using the “rjags” library (Plummer, 2013) in R (R Core Team, 2016).

#### 2.4.1 Specifications of statistical models under Bayesian inference

We used uninformative priors for the MCMC runs: For the treatment intercepts and time slopes, a normal distribution with a mean of 0 and a standard deviation of 0.001 was used, and for the (random) individual intercepts, we used a normal distribution with a mean of 0 and a standard deviation which was randomly drawn from a uniform distribution between 0 and 100. The treatment precisions (in the Gaussian models), the observation precisions (in Poisson's models with overdispersion), and individual precisions (random intercepts for individual) were specified as the inverse of a uniform distribution between 0 and 100. We specified four Markov chains with 200,000 samples each from which the first 100,000 iterations were discarded as an adaptation phase. From the remaining 100,000 posterior samples in each of the four chains, every 20th value was retained to save disk space; this resulted in a total of 20,000 final posterior samples per model. We assessed model convergence by visually checking trace plots of the Markov chains and by applying the Gelman and Rubin's convergence diagnostic (values below 1.1 were accepted). Model performance was checked visually through posterior density plots (only unimodal distributions without shoulders were accepted).

#### 2.4.2 Interpretation of model results under Bayesian inference

Bayesian analyses result in posterior distributions for every model parameter, and this subsequently allows us to deduce the significance of differences in intercepts and slopes between controls and all distances in the MeJA transects in all years. We assessed this significance by subtracting the respective control posterior distributions from their corresponding MeJA posterior distributions and calculating the ratio of the resulting values below and above zero. Positive numbers mean that MeJA‐treated ramets had a higher value for the respective variable or had a greater time slope value than did control ramets. Negative numbers mean that MeJA‐treated ramets had lower values for the respective variable or had smaller time slope values than did the control ramets.

## 3 RESULTS

### 3.1 Inducible defense responses

**Figure 3:**
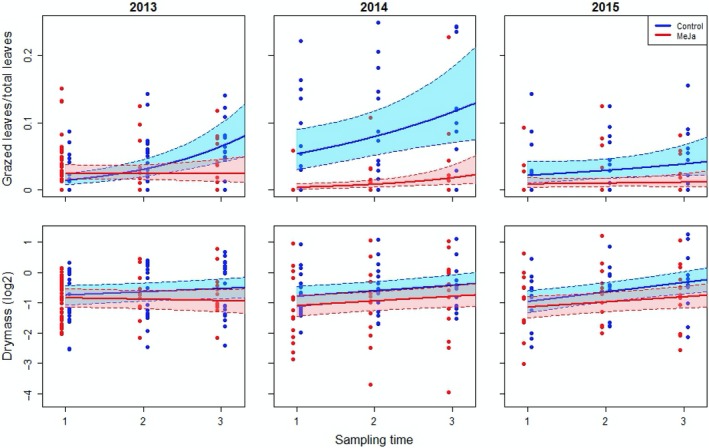
Development in time slope of insect herbivory (ratio of grazed leaves) and growth (dry mass) of bilberry for methyl jasmonate‐treated (dist. 0) and control ramets. Data points were jittered around the three sampling times (6 June, 7 July and 17 August) in order to promote readability of the plots [Colour figure can be viewed at http://wileyonlinelibrary.com]

Methyl jasmonate‐treated ramets showed significantly less insect herbivory than control ramets through the growth season in the first year (slope effect; Table 1; Figure 3), as well as on average (intercept effect and absence of slope effect; Table 1) in the following years, with a particularly strong effect of about ten times fewer grazed leaves in 2014 (Table 1). In 2014 and 2015, there was no significant reduction in insect herbivory through the growth season for the MeJA‐treated ramets (slope effect; Table 1; Figure 3). We did not observe differences in the number of browsed shoots by large herbivores between MeJA‐treated and control ramets in 2013 or 2014, but in 2015, MeJA treatment resulted in four times fewer browsed shoots compared to the control (negative intercept effect; Table 1). In 2013, the growth of untreated bilberry ramets (control) was slightly higher compared to MeJA‐treated ramets (marginally significant slope effect; Figure 3; Table 1), but in 2014 and 2015, we did not find any growth differences between control and MeJA‐treated ramets (Table 1). The numbers of flowers and berries did not differ between MeJA‐treated and untreated ramets in 2013 or 2014 (Table 1). However, 2 years after treatments (2015), MeJA‐treated ramets carried 2.6 more flowers and three times as many berries on average, in comparison with untreated control ramets (Table 1).

**Table 1:** Effects of methyl jasmonate (MeJA) on inducible defense responses in treated bilberry ramets over three consecutive years

	2013		2014		2015	
	Intercept	Slope	Intercept	Slope	Intercept	Slope
Insect grazed leaves	0.56	−0.79**	−2.67**	0.35	−0.92(*)	−0.16
Browsed shoots	−0.10	−0.14	−0.13	−0.10	−1.97**	0.60*
Dry mass	−0.09	−0.15(*)	−0.30	−0.030	−0.18	−0.15
Flowers	−0.37	NA	−0.39	NA	1.71(*)	NA
Berries	−0.28	NA	−0.29	NA	1.87*	NA

Notes

This table shows the effect values of MeJA on intercepts (mean) and time slopes for the MeJA‐treated ramets in relation to the control at distance 0 over three consecutive years. Effects on the intercept reflect general differences on average in each year. Effects on the slope reflect differences in the temporal development during the respective season. Positive numbers mean that MeJA‐treated ramets had higher values for the respective variable or had a higher time slope than the controls. Negative numbers mean that MeJA‐treated ramets had lower values for the respective variable or had lower time slope than the controls. For reproduction variables (flowers and berries), seasonal slopes are not applicable (NA) as they are measured once per season, and only annual means per treatment (intercept effect) are reported.

Significance is indicated by: ***<0.001, **<0.01, *<0.05, (*) <0.1.

### 3.2 Ramet interactions

In 2013, we did not find any differences in insect herbivory between MeJA‐treated and control ramets at dist. 1 (10–40 cm; Table 2). However, a significant reduction in insect herbivory was found for ramets at dist. 2 (40–80 cm; *p* < 0.1), dist. 3 (80–150 cm; *p* < 0.1), and dist. 4 (400–530 cm; *p* < 0.05) through the growth season (slope effect; Table 2). In the two following years, we found reduced insect herbivory on ramets at dist. 1, 2, and 3 (10–150 cm; *p* < 0.05) in 2014, and at dist. 1 (10–40 cm; *p* < 0.01) and 3 (80–150 cm; *p* < 0.05) in 2015 (intercept effects; Table 2).

**Table 2:** Effects of methyl jasmonate (MeJA) on inducible plant defense response of untreated neighboring bilberry ramets at different distances over three consecutive years

	dist. 1		dist. 2		dist. 3		dist. 4	
	Interc.	Slope	Interc.	Slope	Interc.	Slope	Interc.	Slope
2013								
Insect grazed leaves	−0.29	−0.28	0.66	−0.55(*)	0.32	−0.51(*)	0.84	−0.60*
Browsed shoots	−0.70*	0.09	−0.76*	0.14	−0.52	−0.03	−0.64(*)	0.12
Dry mass	−0.84**	−0.05	−0.74**	0.06	−0.73*	−0.04	−0.51*	−0.03
Flowers	−1.22(*)	NA	−1.25(*)	NA	−1.60*	NA	−0.20	NA
Berries	−0.82(*)	NA	−1.11*	NA	−1.11*	NA	0.07	NA
2014								
Insect grazed leaves	−1.04*	−0.17	−1.44**	−0.18	−1.35**	−0.17	−0.46	−0.11
Browsed shoots	−1.22*	−0.12	−0.08	−0.49**	−0.70	−0.37(*)	−0.76	−0.16
Dry mass	−1.03***	−0.01	−0.68*	−0.12	−0.63*	−0.03	−0.40	−0.03
Flowers	−0.32	NA	−0.35	NA	−0.06	NA	−0.40	NA
Berries	−0.45**	NA	−0.17	NA	−0.09	NA	−0.14	NA
2015								
Insect grazed leaves	−1.55**	0.48	0.81	0.03	−1.64*	0.05	−0.71	−0.04
Browsed shoots	−2.18***	0.27	−1.50*	0.43(*)	−0.92	−0.09	−1.88*	0.27
Dry mass	−0.90**	−0.21	−0.62*	−0.07	−0.34	−0.38	−0.08	−0.34
Flowers	−0.54	NA	−0.24	NA	−0.84*	NA	−0.11	NA
Berries	−0.40	NA	−0.28	NA	−0.60(*)	NA	−0.08	NA

Notes

This table shows the effect of MeJA on intercepts (mean) and time slopes for untreated neighbor ramets at different distances (experimental transect) in relation to the control at dist. 0 (control transect) over three consecutive years. Effects on intercept reflect general differences on average in each year. Effects on slope reflect differences in the temporal development during the respective season. Positive numbers mean that neighbor ramets at respective distance had higher values for the respective variable or had higher time slope than the controls. Negative numbers mean that neighbor ramets at respective distance had lower values for the respective variable or had lower time slope than the controls. For reproduction variables (flowers and berries), seasonal slopes are not applicable (NA) as they are measured once per season, and only annual means per treatment (intercept effect) are reported.

Significance is indicated by: ***<0.001, **<0.01, *<0.05, (*) <0.1.

We did not find any difference in the number of browsed shoots through the first growing season for any of the ramets in the MeJA transects (no slope effect in 2013; Table 2). However, in 2013 at dist. 1 (10–40 cm; *p* < 0.05), 2 (40–80 cm; *p* < 0.05), and 4 (400–530 cm; *p* < 0.1), we found significantly fewer browsed shoots on average in MeJA transects compared to control transects (intercept effects in 2013; Table 2). In both 2014 and 2015, ramets at distance 1 (10–40 cm) had significantly fewer browsed shoots compared with control ramets (intercept effects in 2014 and 2015; Table 2).

In 2013, we did not find growth differences through the growth season for any of the untreated ramets in the MeJA transects (no slope effect in 2013; Table 2). No slope effect was observed in 2014 and 2015 either (Table 2). However, from the start of the experiment, neighboring ramets in the MeJA transects had significantly smaller dry mass compared to ramets in the control transects (intercept effects in 2013; Table 2). These size differences were maintained in the MeJA transects in both 2014 and 2015 (Table 2).

In 2013, we found significantly fewer flowers and berries in ramets at distances 1–3 (10–150 cm; *p* < 0.01 and *p* < 0.05) in the MeJA transects (Table 2). In 2014, only ramets at distance 1 (10–40 cm; *p* < 0.01) had significantly fewer berries compared to control ramets (intercept effect in 2014; Table 2), whereas in 2015 only ramets at distance 3 (80–150 cm) had significantly fewer flowers (*p* < 0.05) and berries (*p* < 0.1) compared to control ramets (intercept effects in 2015; Table 2).

## 4 DISCUSSION

We found significant changes in herbivore resistance, growth, and reproduction after MeJA application to wild bilberries under natural environmental conditions over three consecutive seasons. Induced ramets showed significant reductions in growth and herbivory, indicating an efficient strategy of allocating resources from growth to defense over the growing season in the first year (prediction I). Moreover, our findings also show that this state of defense varies in strength and persists across subsequent years (multiannual effect). As predicted, there was a delay of 1 year for the strongest resistance effect to insect herbivores in the MeJA‐treated ramets (prediction III). However, this delayed effect was even longer (2 years) for resistance to herbivory by large animals. In the context of plant–plant interactions, our results indicate long‐distance signaling transfer related to defense between MeJA‐treated ramets and its untreated neighbors under natural conditions (prediction II). The effects of this signaling process on untreated neighboring ramets were multiannual, and its highest effect was delayed, consistent with the patterns found on the MeJA‐treated ramets (prediction III).

### 4.1 Inducible defense responses

The responses related to plant growth (dry mass) and insect herbivory to MeJA application in the first year (2013) suggest that treated plants rapidly allocate resources from growth to defense. These results corroborate previous studies showing that MeJA application on bilberry ramets reduces insect and mammalian herbivory at the expense of growth and reproduction (Hegland et al., 2016; Seldal et al., 2017).

Insect herbivory was significantly reduced in the MeJA‐treated ramets for two subsequent growing seasons (2014 and 2015), indicating a multiannual defense response in bilberry. This multiannual allocation of resources from growth to defense after MeJA treatment in 2013 may explain the lack of a seasonal reduction in insect herbivory in 2014 and 2015 because these plants may already be in a state of “alert” from the previous year. Consistent with our prediction (III), the largest effect in resistance to insect herbivory was found 1 year after the treatment (2014), where MeJA‐treated ramets exhibited about ten times fewer insect grazed leaves compared to controls, followed by reduced herbivory 2 years after treatment (2015). For long‐lived plants, the defense system can be active across multiple growing seasons, a phenomenon referred to as “delayed induced resistance,” depending on the life history of the plant and previous grazing pressure (Haukioja et al., 1985; Zvereva et al., 1997). Induced plant defense responses can persist over a large range of time intervals from rapid (e.g. a few hours or days) to annually delayed induced responses (Agrawal, 1999; Karban & Baldwin, 1997). A study of MeJA‐treated Norway spruce (*Picea abies*) showed less bark beetle colonization and higher terpene content soon after treatment and a relaxation of the defense in the next growing season (Erbilgin, Krokene, Christiansen, Zeneli, & Gershenzon, 2006). Conversely, studies of deciduous trees report more delayed defense responses, which can last for years after the induction (Haukioja, 1982; Haukioja, Ruohomäki, Senn, Suomela, & Walls, 1990; Neuvonen, Haukioja, & Molarius, 1987; Schultz & Baldwin, 1982; Tuomi, Niemelä, Haukioja, Sirén, & Neuvonen, 1984; Valentine, Wallner, & Wargo, 1983). In a meta‐study, Nykänen and Koricheva (2004) showed that induced defense responses in woody plants have the strongest negative impact on the performance of the next generation of herbivores, suggesting a strong delayed defense response in such species.

Two years after treatment (2015), we found reduced herbivory by large mammalian herbivores, where MeJA‐treated ramets had on average four times fewer browsed shoots compared to control ramets. These results suggest that there is a long‐term buildup of defense against large vertebrate herbivores such as red deer, which are abundant in the study area. Generally, rapidly induced plant defenses affect the performance of short‐lived invertebrate herbivores, whereas delayed induced defense responses affect the next generation of short‐lived invertebrate and long‐lived vertebrate herbivores (Haukioja & Hanhimäki, 1985). In addition, delayed induced resistance involves “quantitative defenses,” which are effective against both specialists and generalists, in contrast to rapid induced resistance which involves “qualitative defenses” that are more efficient against generalists but not specialists (Rhoades, 1979). Although costly, quantitative defenses provide better protection against specialized and polyphagous herbivores because they act in a dosage‐dependent manner (Price, Denno, Eubanks, Finke, & Kaplan, 2011). According to the plant apparency theory, plants that are easily found by herbivores, such as trees and shrubs, should invest heavily in quantitative defenses that are effective against a broad spectrum of herbivores (Feeny, 1976). Bilberry is an “apparent” deciduous shrub which store reserves of carbon in stems and roots, enabling it to produce quantitative carbon‐based defenses (e.g. flavonoids and tannins), which are efficient against specialist mammalian herbivores present in the boreal forest, such as red deer (Gallet, 1994). As a result, we hypothesize that bilberry plants may use multiple induced defensive tactics against herbivores: Some are rapidly induced and more efficient against insect herbivores (likely qualitative defenses), while others are delayed induced and more efficient against mammalian herbivores (likely quantitative).

We found that the MeJA treatment led to a reduction in growth (dry mass) in the year of treatment (2013). In contrast to our last prediction (III), this allocation of resources from growth to defense was not significant in the years following treatment. Interestingly, 2 years after treatment (2015), the numbers of flowers and berries of MeJA‐treated plants increased significantly (2.6 and three times, respectively) compared to control plants, suggesting that the defense system reduces herbivory and increases long‐term reproductive success of bilberry. These results indicate that defense mobilization in bilberry lasts for years and thus increases the fitness of defended plants. Although jasmonate‐induced responses function as defenses, this is considered costly for the plant as it has to allocate important resources from growth, reproduction, or other functions. Therefore, as inducible defense responses are considered to be a cost‐saving strategy, plants have the capacity to time the production of these chemicals according to the current environmental conditions, and hence avoid using resources on defenses when they are not needed (Baldwin, 1998). Taking into account the existing competition for limited resources in the boreal forest system, as well as considering the relatively low MeJA effect on treated plants after 2 years, we suggest that induced bilberry plants used a cost‐saving strategy in 2015 by foregoing the excessive costs to allocate resources from reproduction to defenses when these are considered ecologically unnecessary.

### 4.2 Multiannual ramet interactions

Untreated bilberry ramets growing at distances of between ten centimeters to five meters from MeJA‐treated ramets showed reduced insect herbivory compared to untreated control ramets. These findings indicate that the MeJA itself or the emission of VOCs from MeJA‐treated ramets can activate the defense system of untreated neighbor ramets at distances of up to five meters. Our results are consistent with results from studies involving other species where above‐ and belowground signaling activate the defense system and reduce herbivory of untreated neighbor plants (Baldwin, Kessler, & Halitschke, 2002; Dicke & Bruin, 2001; Heil & Karban, 2010; Pickett, Rasmussen, Woodcock, Matthes, & Napier, 2003).

Bilberry has interconnected ramets with extensive belowground rhizomes (Tolvanen & Laine, 1997). Therefore, both airborne and belowground signaling probably contributed to the activation of the defense system of untreated ramets in this study (Chen, Lei, & Liu, 2011; Gómez, Latzel, Verhulst, & Stuefer, 2007; Gomez & Stuefer, 2006; Gómez et al., 2010). Regardless of the type of signaling strategy, the evidence here and in other studies supports two types of responses by the neighboring “eavesdropping” plants: The induction of a direct defense mechanisms that makes them resistant to subsequent herbivory (e.g. altering palatability and/or toxicity of leaf tissues) and an indirect defense strategy, such as the recruitment of natural enemies as “bodyguards” (Dicke, Agrawal, & Bruin, 2003).

The elevated resistance to herbivory of untreated neighbor ramets lasted for several growth seasons, although this varied in time and space. In 2013, we found reduced insect herbivory of untreated ramets growing at distances of up to five meters from the MeJA‐treated ramets. In the subsequent growing season (2014), however, only ramets growing close to the MeJA‐treated ramets showed less insect herbivory compared to control ramets. Consistent with the results found among the MeJA‐treated ramets, untreated neighbor ramets were most resistant to herbivory in 2014. Two years after the MeJA treatment (2015), the effects of the defense system started to relax in some of the neighbor ramets at greater distances from the MeJA‐treated ramet. Previous studies that resurrected interplant communication in the last decade have shown similar results by conducting laboratory and field experiments and exploring molecular, physiological, and ecological data. For instance, Dolch and Tscharntke (2000) demonstrated that experimental defoliation of single trees in different sites in Germany caused natural herbivory to increase with distance from the defoliated tree, and the authors attributed this effect to above‐ or belowground signaling. Similar to our study, another field experiment conducted over three consecutive years showed that experimentally damaged sagebrush plants led to herbivory resistance in neighboring tobacco plants compared to those neighboring undamaged sagebrush (Karban et al., 2000). This plant–plant interaction process was correlated with induced emissions of MeJA in damaged sagebrush and increased production of an important defense chemical (polyphenol oxidase) in the neighboring tobacco plants.

An unexpected situation occurred regarding our growth‐related results on untreated neighboring ramets. Untreated neighboring ramets from the experimental transects were significantly smaller than the ramets from the control transects already at the onset of the experiment in 2013 (i.e. significant intercept effect in 2013). Because of this bias in plant size, we cannot imply that the observed differences in dry mass are a result of a trade‐off between growth and defense caused by plant–plant interaction. However, this bias likely remains without any effect for our interpretation, as neither our data or analysis show any signs of differences for dry mass during the season (slope effects) between controls and MeJA neighboring ramets.

We found the strongest resistance against herbivorous insects in untreated neighbor ramets 1 year after the MeJA treatment (2014; Insect grazed leaves; Table 2), where ramets at dist. 1, 2, and 3 (10–150 cm) showed on average four times less insect herbivory compared to control ramets. However, browsing by large mammalian herbivores was lowest in untreated neighbor ramets in 2015, 2 years after the MeJA treatment (2015; Browsed shoots; Table 2). Both results are consistent with the responses of MeJA‐treated ramets in 2014 and 2015 (Insect grazed leaves; Browsed shoots; Table 1). As a result, our findings on untreated neighboring ramets appear to be consistent with the results found for the MeJA‐treated ramets, suggesting that both defensive strategies of induced plants and its neighbors are effectively multiannual and that the strongest effects are delayed.

For inducible resistance to cause cyclic fluctuations in herbivore populations, the intensity of the rapid inducible resistance has to be weaker than the long‐term resistance in the subsequent years (Haukioja & Hanhimäki, 1985). However, as indicated by several authors (Fox, 1981; Högstedt, Seldal, & Breistøl, 2005; Lundberg, Järemo, & Nilsson, 1994; Myers, 1988; Seldal, Andersen, & Högstedt, 1994; Spencer, 2013; Underwood, 1999), more studies of the delayed action of plant defense responses under natural field conditions are necessary to better understand how these systems affect herbivore populations. However, due to the large scale (i.e. space, time) and complexity of ecosystems, such studies are challenging to design (Underwood, 1999). Nevertheless, there are some studies that show close correlations between bilberry production and local population sizes of both insects and large herbivores known to feed on bilberry (Selås, 1997, 2000, 2006; Selås, Kobro, & Sonerud, 2013).

In summary, our findings provide evidence for long‐term effects of plant–plant signaling mediated by jasmonate‐induced responses in bilberry, indicating that induced plants are “good” neighbors due to ecological facilitation with conspecifics under natural conditions. The demonstrated effects of below‐ and aboveground plant–plant interactions, especially related to herbivore resistance, varied in efficacy according to time (seasons after induction) and distance from the induced plant emitting the chemical information to its neighbors. Moreover, the documented multiannual effect and the delay of the highest level of induced resistance on MeJA‐treated and untreated neighbor bilberry ramets may have important implications for our understanding of outbreaks of insect and mammalian herbivore populations in the boreal ecosystem.

## AUTHORS’ CONTRIBUTIONS

T.S., S.J.H., and K.R. conceived the initial ideas and designed methodology and collected the data; J.P.T. and R.F.B analyzed the data; R.F.B led the writing of the manuscript. All authors contributed equally to the drafts and gave final approval for the publication.

## DATA ACCESSIBILITY

All the data generated for this study are publicly available at Dryad Digital Repository.
